# Impact of Oral Rinsing with Octenidine Based Solution on SARS-CoV-2 Loads in Saliva of Infected Patients an Exploratory Study

**DOI:** 10.3390/ijerph19095582

**Published:** 2022-05-04

**Authors:** Ralf Smeets, Susanne Pfefferle, Henning Büttner, Johannes K. Knobloch, Marc Lütgehetmann

**Affiliations:** 1 Department of Oral and Maxillofacial Surgery, University Medical Center Hamburg-Eppendorf (UKE), 20251 Hamburg, Germany; 2 Department of Oral and Maxillofacial Surgery, Division of Regenerative Orofacial Medicine, University Medical Center Hamburg-Eppendorf (UKE), 20251 Hamburg, Germany; 3 Center for Diagnostics, Institute of Medical Microbiology, Virology and Hygiene, University Medical Center Hamburg-Eppendorf (UKE), 20251 Hamburg, Germany; s.pfefferle@uke.de (S.P.); h.buettner@uke.de (H.B.); j.knobloch@uke.de (J.K.K.)

**Keywords:** SARS-CoV-2, octenidine, oral rinsing

## Abstract

Objective: In this study, the in-vivo effect of an antiseptic mouth rinse with Octenisept plus phenoxyethanol (OCT + PE) on the oral SARS-CoV-2 load was investigated. Material and Methods: In eight COVID-19 patients, saliva samples were obtained before mouth rinsing and at five time points post rinsing with OCT + PE (*n* = 47 saliva samples in total). SARS-CoV-2 RNA was detected and quantified by RT-qPCR and virus isolation in cell culture was performed to assess for infectivity. Results: Immediately after mouth rinsing (1 min), a significant reduction of the SARS-CoV-2 RNA loads in saliva was achieved (*p* = 0.03) with 7/8 participants having SARS-CoV-2 RNA levels undetectable by RT-qPCR. At later time points, RNA levels returned to baseline levels in all study participants. Infectivity of saliva samples was demonstrated by successful virus isolation from saliva samples collected at later time points. Conclusions: This study highlights that saliva samples from COVID-19 patients are infectious and demonstrates that mouth rinsing with OCT + PE temporarily leads to a significant reduction of the SARS-CoV-2 load in saliva. Clinical relevance: Mouth rinsing with OCT + PE could provide a simple, rapid, and efficient method for SARS-CoV-2 infection prevention, particularly in the field of dental and respiratory medicine

## 1. Introduction

The pandemic coronavirus SARS-CoV-2 can be transmitted via direct or indirect contact with infected individuals through aerosol formation, saliva, respiratory secretions, or respiratory droplets released by coughs, sneezes, talking, or singing [1]. It is shown that transmission occurs in the early phase of the SARS-CoV-2 associated coronavirus disease 2019 (COVID-19) and often before symptom onset [2], thus posing a major challenge for disease prevention and infection control measures. Accordingly, healthcare workers in the field of dental and respiratory medicine, such as dentists, maxillofacial surgeons, and ENT physicians are at high risk for SARS-CoV-2 transmission [3,4]. Besides personal protective equipment, safety precautions, and hand hygiene, pre-procedural antiseptic oral rinsing immediately before oral care procedures have been recommended by various health authorities worldwide [4,5,6,7]. Recently published in vitro experiments reveal that octenidine dihydrochloride (OCT) plus phenoxyethanol (PE) or povidone-iodine (PI) reduce infectious SARS-CoV-2 within 30 s by more than 4 log_10_ [8,9,10], thus indicating a >10,000-fold reduced infectivity of virus-containing supernatants after incubation with the substances. OCT belongs to the group of bispyridines and displays activity against bacteria, fungi, and enveloped viruses. Th eaddition of PE leads to a fast onset of action after 15 s. However, current recommendations are mainly based on the general ability of the various compounds to disrupt lipid membranes of pathogens and thus assumed effectiveness against enveloped viruses such as SARS-CoV-2. The *World Health Organization* (WHO) and Chinese health authorities recommend the use of hydrogen peroxide (H_2_O_2_) or 0.2% PI. The *Center for Disease Control and Prevention* (CDC) recommend chlorhexidine digluconate (CHX), Cetylpyridinium chloride (CPC), or PI and essential oils [11]. German health authorities suggest gargling with 0.2% PI before dental treatment [12,13], while the *German Working Group for Hygiene in Dentistry* (Deutscher Arbeitskreis für Hygiene in der Zahnmedizin, DAHZ) states that OCT-based rinses can be used [5]. 

The aim of the present study in COVID-19 patients was to analyze the in-vivo antiviral effect in the saliva by oral rinsing with a commonly used OCT-based antiseptic rinsing solution. Viral RNA quantification in saliva samples before and after oral rinsing and assessment of infectivity by virus isolation experiments are performed.

## 2. Materials and Methods

### 2.1. Study Design, Ethics, and Patients

This exploratory study evaluates the short-term effect of rinsing the oral cavity with commonly used OCT plus PE (OCT + PE) based antiseptic rinsing solution (octenisept^®^ Schülke & Mayr, Norderstedt, Germany) to reduce the SARS-CoV-2 burden in the saliva. The primary study outcome was SARS-CoV-2 RNA load. The null hypothesis for the primary outcome was the reduction of less than 1 log after mouth-rinsing with OCT plus PE. The secondary study outcome was to analyze infectivity by virus isolation. The inclusion criteria comprised male or female individuals between 18 and 90 years of age with SARS-CoV-2 infection as confirmed by Reverse-Transcription-quantitative Polymerase Chain Reaction (RT-qPCR) within the last 48 h. Exclusion criteria were applied e.g., the inability to understand instructions, inflammation in the oral cavity, respiratory symptoms, or fever at enrolment in the study. 

The study was performed in accordance with the ethical standards of the institutional and/or national research committee and with the 1964 Helsinki Declaration and its later amendments or comparable ethical standards. The study was approved by the Research and Ethics Committee of the Medical Board Hamburg (PV7415). Overall, *n* = 8 patients with active SARS-CoV-2 infection are included in this study. 

### 2.2. Sample Collection

In total, six saliva samples were collected for each participant by spitting into a sterile tube (before rinsing and at time points 1 min, 30 min, 60 min, 240 min, and 360 min after rinsing). 

Subjects were briefed to fast 30 min prior to collection of the initial saliva sample (1.2 mL) and were then instructed to rinse their mouth with 20 mL of the antiseptic rinsing solution for 20 s in accordance with the product information leaflet. Successful adherence to the study protocol was observed and documented by the research physician via ClickDoc Videosprechstunde^®^ (CompuGroup Medical, Koblenz, Germany). 

### 2.3. Molecular Diagnostic 

SARS-CoV-2 RNA in saliva was quantified and detected by qPCR. Briefly, saliva was 1:1 diluted using Cobas PCR media (Roche) and samples were loaded on the fully automated cobas6800/8800 system (Roche, Mannheim, Germany) using the IVD SARS-CoV-2 Cobas PCR assay (see also [14,15]). Standard SARS-CoV-2 RNA reference material (obtained from INSTAND e.V., Düsseldorf, Germany) was used for quantification. To calculate log_10_ RNA copies/mL (y) based on ct-values, the following targets and conversion formulae were used y = −0.308x + 13.81 (cobas SARS-CoV-2, target T2). To analyze the potential interference of Octenidin rinsing solution with the quantitative SARS-CoV-2 RNA detection, we performed a method comparison experiment. Briefly, a highly positive SARS-CoV-2 RNA saliva sample diluted in either SARS-CoV-2 negative saliva or SARS-CoV-2 negative saliva + 5% octenident to generate two dilution series with 6 levels (1:10) and 5 repeats at each dilution level covering the whole linear range (<10^3^ to 10^7^ copies/mL), samples were analyzed by the IVD SARS-CoV-2 Cobas PCR assay. For method comparison, Ct values (*n* = 60; target 2: E-gene) were analyzed. The mean bias over the whole range was 0.13 Ct. Non parametric Passing-Bablok regression analysis (samples +/− octenidin solution) showed high correlation r^2^ = 0.991 with a slope of y = 0.364 + 0.99 * x). These results indicate that the octenident solution does not interfere with the quantitative qPCR detection of SARS-CoV-2 RNA in saliva samples.

### 2.4. Cell Culture and Virus Isolation 

Virus isolation experiments were performed for all available samples (*n* = 47 samples in total). For infection, Vero E6 cells (ATCC-CRL-1008) seeded in 24-well tissue culture plates were inoculated with 500 µL of the saliva samples. After 72 h of incubation at 37 °C, supernatants of cultures were harvested and virus growths were quantitatively assessed by SARS-CoV-2 Cobas PCR assay. An increase of at least 1log SARS-CoV-2 RNA compared to baseline viral load was used to identify samples with successful virus isolation [16].

### 2.5. Statistic 

Statistical analysis was performed using Graphpad 7 (San Diego, CA, USA), Rstudio v1.4.1103 and Validation manager software V 2022.3.3 (Finbiosoft, Espoo, Finland). 

## 3. Results

### 3.1. SARS-CoV-2 RNA Quantification 

**Figure 1:**
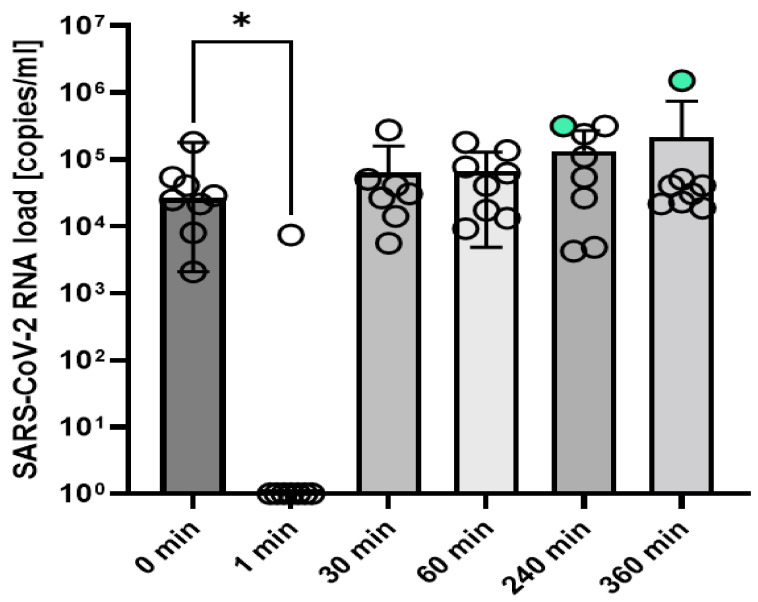
Octenidine dihydrochloride (OCT) plus phenoxyethanol effectively reduces detectable viral RNA in the saliva of SARS-CoV-2 positive individuals. SARS-CoV-2 RNA levels in the saliva of 8 subjects as quantified by RT-qPCR pre-rinsing with OCT plus phenoxyethanol (0 min) and 1, 30, 60, 240 and 360 min after rinsing with OCT plus phenoxyethanol are illustrated. RNA levels < LoD were set to 1 × 10^0^ copies/mL to allow for logarithmic presentation. Median RNA levels for each time point and 95% CI are indicated. Significant differences are indicated by asterisk (* = *p* < 0.05, unpaired *t*-test). Circles represent individual participant values with circles highlighted in light turquoise represent infectious samples as proved by successful virus isolation.

In the initial pre-mouthwash samples of the RT-qPCR positive participants (*n* = 8), a median SARS-CoV-2 RNA level of 2.68 × 10^4^ copies/mL (range 2.09 × 10^3^–1.81 × 10^5^ copies/mL) was detected. One minute after mouth rinsing with OTC + PE containing solution, SARS-CoV-2 RNA levels significantly dropped compared to the initial RNA levels (unpaired *t*-test, *p* = 0.031, see Figure 1). At this time point, SARS-CoV-2 RNA levels were below the limit of detection (LoD) of the RT-qPCR in 7/8 (87.5%) participants (median SARS-CoV-2 RNA level < LoD, range 0–7.47 × 10^3^ copies/mL). In one participant, only a slight SARS-CoV-2 RNA reduction of 66% compared to the initial RNA level was observed at 1 min after rinsing, in this participant, the lowest detected RNA level was 7.43 × 10^3^ copies/mL. 

SARS-CoV-2 RNA levels in the saliva of 8 subjects as quantified by RT-qPCR pre-rinsing with OCT plus phenoxyethanol (0 min) and 1, 30, 60, 240, and 360 min after rinsing with OCT plus phenoxyethanol are illustrated. RNA levels < LoD were set to 1 × 100 copies/mL to allow for logarithmic presentation. Median RNA levels for each time point and 95% CI are indicated. Significant differences are indicated by asterisk (* = *p* < 0.05, unpaired *t*-test). Circles represent individual participant values with circles highlighted in light turquoise representing infectious samples as proven by successful virus isolation. 

**Figure 2:**
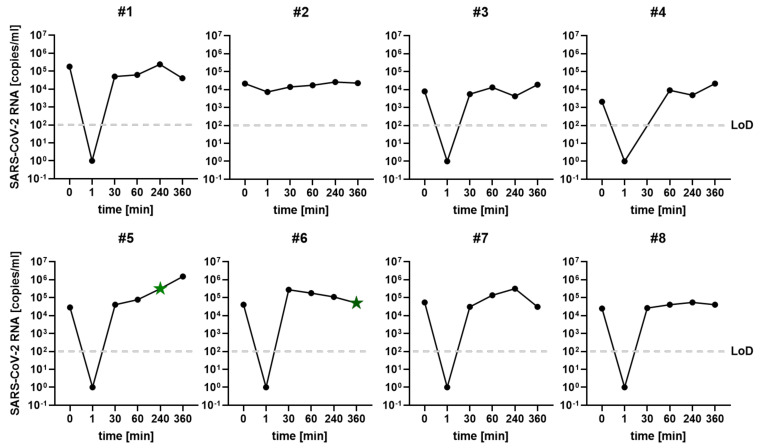
SARS-CoV-2 RNA kinetics of the included 8 subjects: SARS-CoV-2 RNA load [copies/mL] in saliva samples at the analyzed time points is illustrated. The dashed light grey line corresponds to the limit of detection of the RT-qPCR used [14]. The points marked with a green asterisk (time points 240 h subject #5, 360 h subject #6) correspond to infectious samples from which virus isolation was successful.

At 30 min after rinsing, SARS-CoV-2 RNA could again be detected in the saliva of all participants with available samples of that time point (7/8, one sample missing). The median SARS-CoV2 RNA level at this time point was 3.08 × 10^4^ copies/mL (range 5.63 × 10^3^–2.77 × 10^5^ copies/mL). In 4 of these 7 participants (57%), the RNA levels at 30 min after rinsing were below the initial value before the mouth rinse. At all later time points after rinsing, SARS-CoV-2 RNA levels in the saliva samples of all participants were in the range of the baseline RNA levels and remained at stable levels until the end of the observation period (see Figure 1, for individual kinetics refer to Figure 2).

### 3.2. Infectivity of the Saliva Samples

To determine infectivity, virus isolation in cell culture was attempted from all saliva samples of the 8 RT-qPCR positive participants. Overall, *n* = 47 samples collected at six time points were assessed (one sample of time point 30 min after rinsing was missing). Infectious virus was successfully isolated from 2/47 (4%) samples. Both infectious samples were obtained at late time points (at 240 min and 360 min after mouth rinsing). SARS-CoV-2 RNA levels in the infectious samples were 3.19 × 10^5^ and 5.06 × 10^4^ copies/mL, respectively (see Figure 1 and Figure 2 for individual courses). 

## 4. Discussion

### 4.1. Based on the Study Results, the Null Hypotheses for the Primary Outcome (SARS-CoV-2 RNA Reduction of Less than 1 Log) by Oral Rinsing with OCT plus Phenoxyethanol Was Rejected

The implementation of oral rinsing as a preventive method to reduce SARS-CoV-2 levels in the saliva of patients, and thus protect health care workers from possible virus transmission, is recommended by national and international health authorities [4,5,6,7,11,12,13]. However, clinical evidence and in-vivo data are lacking. 

In the present study, we provide evidence that one of the commonly used substances for oral rinsing in the health sector (OCT plus PE), leads to a rapid and effective reduction of SARS-CoV-2 RNA levels in the saliva of the user. We were able to show that the viral RNA levels drop significantly (*p* = 0.03) in the saliva within the first minute after the mouth rinse and in almost all participants (7/8). Notably, while most participants responded very uniformly to the substance, in one participant, only a minor SARS-CoV-2 RNA reduction in the saliva was observed after rinsing. This phenomenon cannot be conclusively explained and the possibility of incorrect application or ineffectiveness of the substance arises. However, it is conceivable that SARS-CoV-2 containing material from the deep respiratory tract of that participant (e.g., coughed up or entering the oral cavity through sneezing) may have falsified an OCT + PE effect that was actually present. 

The fast onset of OCT + PE effects (<1 min) as demonstrated here in the saliva is relevant for pre-procedural application in clinical practice, e.g., in dentists’ or oral and maxillofacial surgeons’ offices, and represents an advantage over other oral rinses tested in-vivo so far with longer, unreliable times until onset of effects [17,18]. 

In this study, we assessed the infectivity of the saliva samples by cell culture experiments and were indeed able to prove infectivity for two of the saliva samples obtained at late observation time points underlining the risk of virus transmission. Notably, all initial samples collected before the OCT + PE rinsing were culture-negative. These samples were first examined in RT-qPCR and the virus cultivation was only carried out after the result was obtained, therefore the infectivity in these samples could already have been significantly reduced. However, as only comparatively mildly ill persons were enrolled at a considerable time after their COVID-19 diagnosis, SARS-CoV-2 RNA levels in all samples analyzed were at the lower range of RNA levels in the respiratory tract of severely ill or even asymptomatic patients in the early phase of the disease [2]. Moreover, it is known that the probability of virus isolation decreases with the increasing duration of the disease [2]. Additionally, saliva is not the common material used for virus isolation and it is quite conceivable that besides possibly containing antibodies (IgA, IgG), enzymes contained in saliva may interfere with virus isolation. Nevertheless, we believe that our results with a proof of infectivity only in samples obtained late after mouth rinsing indicate that OCT + PE not only reduces the amount of RNA in the saliva, but is in line with the in-vitro data available [8,9,10], might reduce the burden of SARS-CoV-2 infectious particles in the oral cavity in-vivo. 

### 4.2. Limitations

Despite the promising results of the present study, certain limitations should be noted. Firstly, the study was conducted without a control group. Therefore, it cannot be ruled out that the reduction effects are partly based on a dilution by the mouth rinse. Yet it has been shown that mouth rinsing with tap water has no impact on viral load [19].

Secondly, possibly remaining OCT + PE compound in the saliva sample was not inactivated before the sample was added to cell culture and the low number of individuals may cause statistical bias, hence a larger number of participants in studies is required for valid statements. The study design was an exploratory study therefore power calculation to determine the study’s sample size was not performed. Possible effects of the OCT + PE rinse benefit on clinical outcomes need to be evaluated in future randomized, placebo-controlled clinical trials. Given the early onset effect, OCT + PE might be primarily indicated for interventions of short duration, however, repeated use during prolonged dental procedures might be conceivable and could also be investigated in follow-up studies.

## 5. Conclusions

Results of the present study provide clinical data revealing that OCT + PE might temporarily reduce the SARS-CoV-2 RNA burden in the oral cavity with a rapid onset of effects. Antiseptic oral rinsing with OCT + PE might thus represent a simple and safe intervention to reduce the risk of SARS-CoV-2 transmission, particularly in the field of dental and respiratory medicine, especially during short-lasting examinations or in the initial phase of the procedures, respectively.

Based on the encouraging results, further studies should be conducted to prove the clinical efficacy of the compounds. It must be stated that the transfer of material from the deep airways can mitigate the observed effect. Thus, the use of OCT + PE can be a useful component in infection prevention. However, other infection prevention measures (personal protective equipment for acting personnel) cannot be replaced by mouth rinsing in patients.
